# The proteomic analysis of bovine embryos developed *in vivo* or *in vitro* reveals the contribution of the maternal environment to early embryo

**DOI:** 10.1186/s12864-022-09076-5

**Published:** 2022-12-19

**Authors:** Charles Banliat, Coline Mahé, Régis Lavigne, Emmanuelle Com, Charles Pineau, Valérie Labas, Benoit Guyonnet, Pascal Mermillod, Marie Saint-Dizier

**Affiliations:** 1 INRAE, CNRS, Tours University, IFCE, UMR PRC, Nouzilly, France; 2 Union Evolution, Rue Eric Tabarly, Noyal-Sur-Vilaine, France; 3 Univ Rennes, Inserm, EHESP, Irset (Institut de Recherche en Santé, Environnement et Travail), UMR_S 1085, Rennes, France; 4 Univ Rennes, CNRS, Inserm, Biosit UAR 3480 US_S 018, Protim Core Facility, Rennes, France; 5 Pixanim, INRAE, Tours University, CHU of Tours, Nouzilly, France

**Keywords:** Embryo, Proteomics, Mass spectrometry, Morula, Blastocyst, Cattle, Oviduct, Development

## Abstract

**Background:**

Despite many improvements with *in vitro* culture systems, the quality and developmental ability of mammalian embryos produced *in vitro* are still lower than their *in vivo* counterparts. Though previous studies have evidenced differences in gene expression between *in vivo-* and *in vitro*-derived bovine embryos, there is no comparison at the protein expression level.

**Results:**

A total of 38 pools of grade-1 quality bovine embryos at the 4–6 cell, 8–12 cell, morula, compact morula, and blastocyst stages developed either *in vivo* or *in vitro* were analyzed by nano-liquid chromatography coupled with label-free quantitative mass spectrometry, allowing for the identification of 3,028 proteins. Multivariate analysis of quantified proteins showed a clear separation of embryo pools according to their *in vivo* or *in vitro* origin at all stages. Three clusters of differentially abundant proteins (DAPs) were evidenced according to embryo origin, including 463 proteins more abundant *in vivo* than *in vitro* across development and 314 and 222 proteins more abundant *in vitro* than *in vivo* before and after the morula stage, respectively. The functional analysis of proteins found more abundant *in vivo* showed an enrichment in carbohydrate metabolism and cytoplasmic cellular components. Proteins found more abundant *in vitro* before the morula stage were mostly localized in mitochondrial matrix and involved in ATP-dependent activity, while those overabundant after the morula stage were mostly localized in the ribonucleoprotein complex and involved in protein synthesis. Oviductin and other oviductal proteins, previously shown to interact with early embryos, were among the most overabundant proteins after *in vivo* development.

**Conclusions:**

The maternal environment led to higher degradation of mitochondrial proteins at early developmental stages, lower abundance of proteins involved in protein synthesis at the time of embryonic genome activation, and a global upregulation of carbohydrate metabolic pathways compared to *in vitro* production. Furthermore, embryos developed *in vivo* internalized large amounts of oviductin and other proteins probably originated in the oviduct as soon as the 4–6 cell stage. These data provide new insight into the molecular contribution of the mother to the developmental ability of early embryos and will help design better *in vitro* culture systems.

**Supplementary Information:**

The online version contains supplementary material available at 10.1186/s12864-022-09076-5.

## Background

The high frequency of embryo demise after *in vitro* development is a general feature in mammalian species. Recent data registered by the European Society of Human Reproduction and Embryology (ESHRE) indicate that the rate of clinical pregnancy after transfer of *in vitro*-fertilized human embryos averages 35% over 39 countries [1]. In cattle, the number of *in vitro*-produced embryos that are transferred worldwide is continuously growing and has overpassed that of *in vivo*-produced embryos since 2016 [2]. The main objectives of embryo production and transfer in cattle is to disseminate genetically superior females and optimize animal breeding for milk and meat production. Data collected by the International Embryo Transfer Society (IETS) indicate that more than 1.2 million cattle embryos are transferred worldwide each year, 80% of which are produced *in vitro *[3]. However, despite many improvements in *in vitro* systems and embryo culture media, the blastocyst yield and embryo quality did not significantly progress over the past decades. On average, 20% to 40% of cultured presumptive zygotes reach the blastocyst stage, and many of these are unable to sustain development following embryo transfer [2, 4]. Based on studies reported over the past 25 years, the pregnancy rates of recipient cows carrying *in vitro*-produced (IVP) embryos are 10 to 40% lower when compared with *in vivo*-derived embryos generated by superovulation, and only 27% of cows receiving IVP embryos will produce a living calf [5]. Furthermore, the ability of cattle embryos to survive after cryopreservation is much lower after *in vitro* development than *in vivo* development [2, 4]. Bovine and human early embryos are similar in terms of biochemical regulatory processes, transcriptomic dynamics, and the kinetics of development up to the blastocyst stage [6–8], reinforcing interest in cattle embryos as models to study the contribution of the maternal environment to embryo quality.

Earlier studies comparing bovine embryos developed *in vitro* or *in vivo* have reported differences in morphology and ultrastructure [9–12], lipid profiles [13], and energy metabolism [14–16]. Furthermore, transcriptomic studies comparing bovine embryos developed *in vitro* or *in vivo* have reported differences in gene expression [17–20] and methylation patterns [21], revealing molecular mechanisms underlying the higher developmental ability of *in vivo*-developed embryos. Based on the previously reported “quiet embryo” theory, it was hypothesized that at the time of embryonic genome activation (between the 8-cell and 16-cell stages), *in vivo*-derived embryos may have a lower activation of protein synthesis and slightly lower metabolic activity compared to their *in vitro* counterparts, increasing their chance to pursue development [22–24]. In addition, based on our previous work, 56 proteins, including oviduct-specific glycoprotein 1, were internalized by *in vitro-*produced embryos during incubation with oviduct fluid [25] so we hypothesized that those proteins would be at higher abundance in *in vivo-* compared to *in vitro*- derived embryos. However, due to the difficulty to access early embryos *in vivo* and scarcity of materials, the proteomic comparison between *in vivo*- and *in vitro*-derived embryos in mammals remained unexplored. In a previous study, we analyzed the proteomic dynamics of bovine *in vivo*-derived embryos from the 4–6 cell to blastocyst stages [26]. In the present study, the same pools of *in vivo*-developed embryos were compared to contemporary pools of bovine embryos produced *in vitro*. All embryos were analyzed in the same analysis batch by nanoliquid chromatography coupled with tandem mass spectrometry (nanoLC-MS/MS) and label-free quantification.

## Results

Only embryos of grade-1 quality, i.e., of high morphological quality, with no sex sorting, were included in the analysis. Although not systematically observed, *in vitro*-produced embryos tended to appear darker than those collected *in vivo* (see representative pictures in Figure S1).

### Distribution of proteins identified between *in vivo* and *in vitro*-derived embryos

**Fig. 1:**
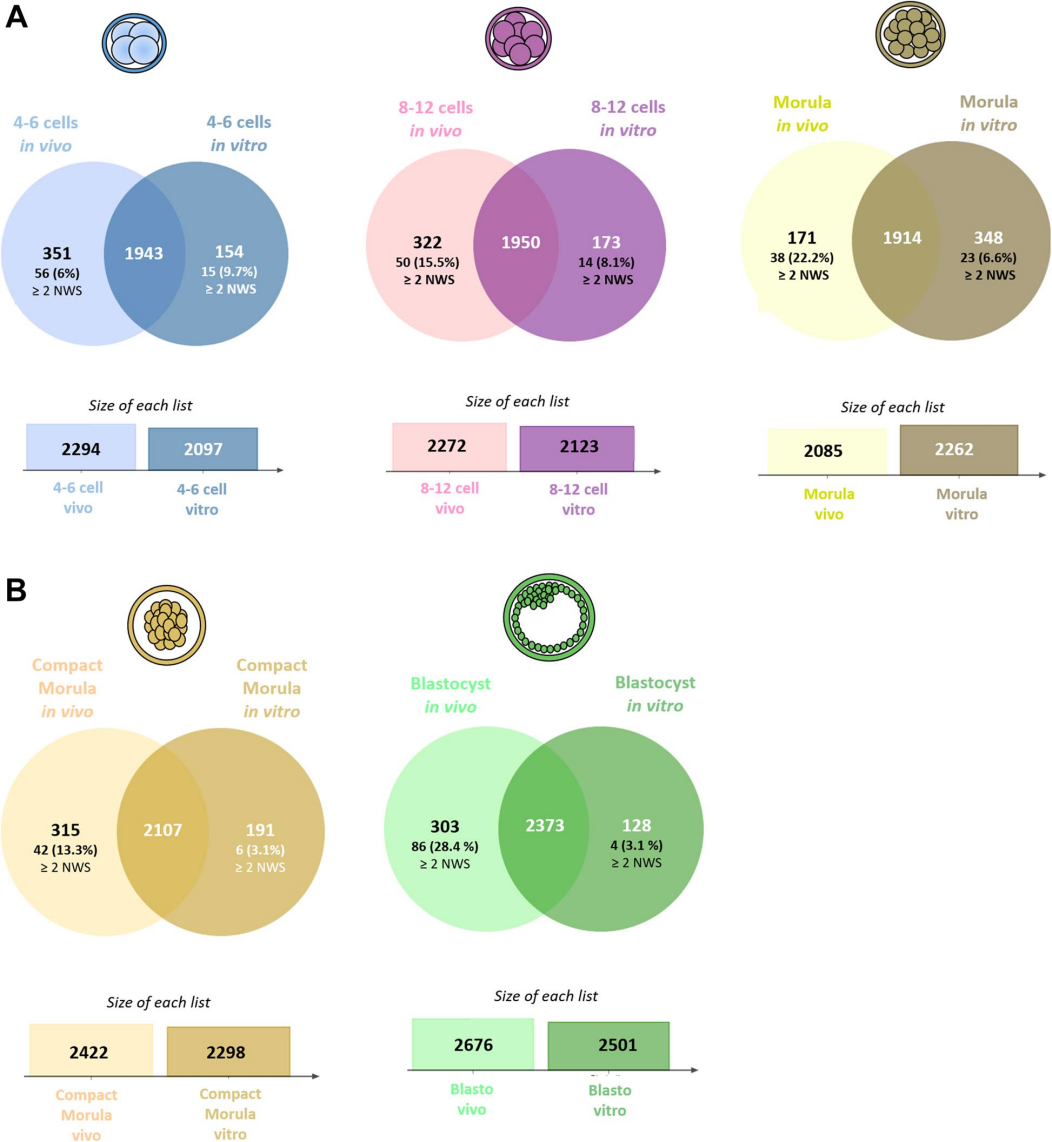
Comparative analysis of proteins identified in bovine early embryos produced *in vivo* or *in vitro*. The Venn diagram indicates the overlap between origins at each stage, and the histograms in the bottom indicate the number of proteins identified in each pool of embryos

A total of 3,028 proteins were identified by at least two unique peptides in embryos (false discovery rate < 0.01%; see Table S1 for the complete list of proteins with their accession number, gene symbol, and normalized quantification). Figure 1 shows the number and distribution of identified proteins between *in vivo* and *in vitro*-derived embryos at each stage. An increase in the number of identified proteins was observed from the 4–6 cell to blastocyst stages in both groups of embryos (+ 16.6% and + 19.3% in *in vivo* and *in vitro*-derived embryos, respectively). Overall, more than 85% of proteins were shared between *in vivo* and *in vitro*-derived embryos (see Table S1 for the list of specific proteins at each stage). Most of proteins detected exclusively *in vivo* or *in vitro* were at low abundance as only 3%–28% were quantified with more than two normalized weighted spectra (NWS; Fig. 1).

### Global analysis of differentially abundant proteins between *in vivo* and *in vitro*-derived embryos across development

**Fig. 2:**
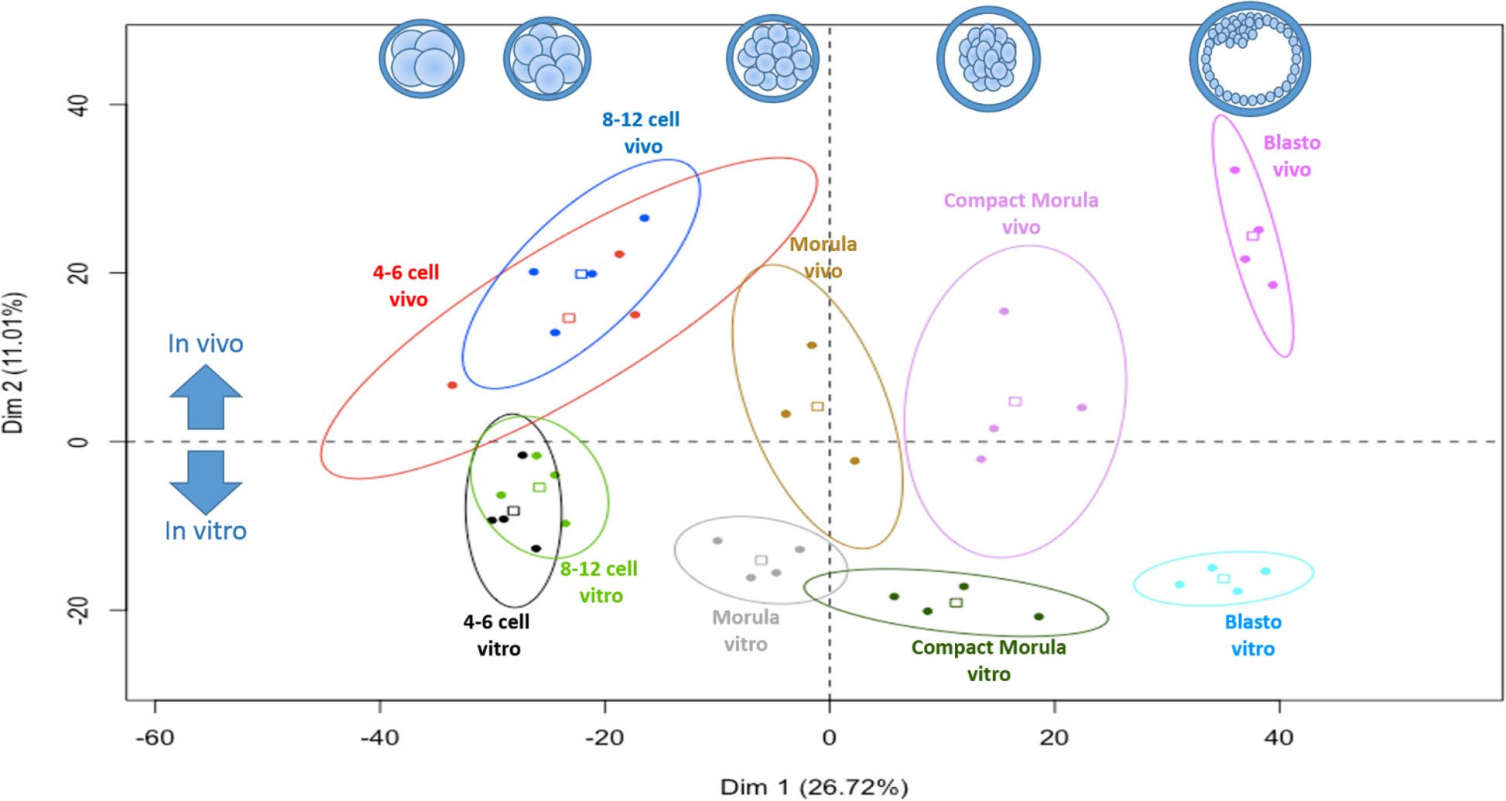
Principal component analysis of all embryo pools from the 4–6 cell to blastocyst stages. The 2,186 proteins quantified with a minimum of 2 normalized weighted spectra in at least one condition were considered. Scatter plots represent the position of each pool of embryos along the first two principal components. The variability between pools was mainly explained by their stage of development on the first horizontal dimension (Dim 1; 26.7% of variance) then by their origin (*in vivo* vs. *in vitro*) on the second vertical dimension (Dim 2; 11.0% of variance). The square in each ellipse represents the mean of data for a given condition, and colored ellipses represent the 95% confidence intervals

A total of 2,184 proteins quantified with a minimum of 2 NWS in at least one condition were retained for statistical analysis (Table S1). Figure 2 shows the principal component analysis (PCA) of all quantified proteins. The first dimension of PCA on the horizontal axis separated embryo pools according to their stage of development, except between the 4–6 cell and 8–12 cell stages, which clustered together. The second dimension of PCA on the vertical axis showed a clear separation of embryo pools according to their origin, starting at the 4–6 cell stage, with maximal gap at the blastocyst stage (Fig. 2).

**Fig. 3:**
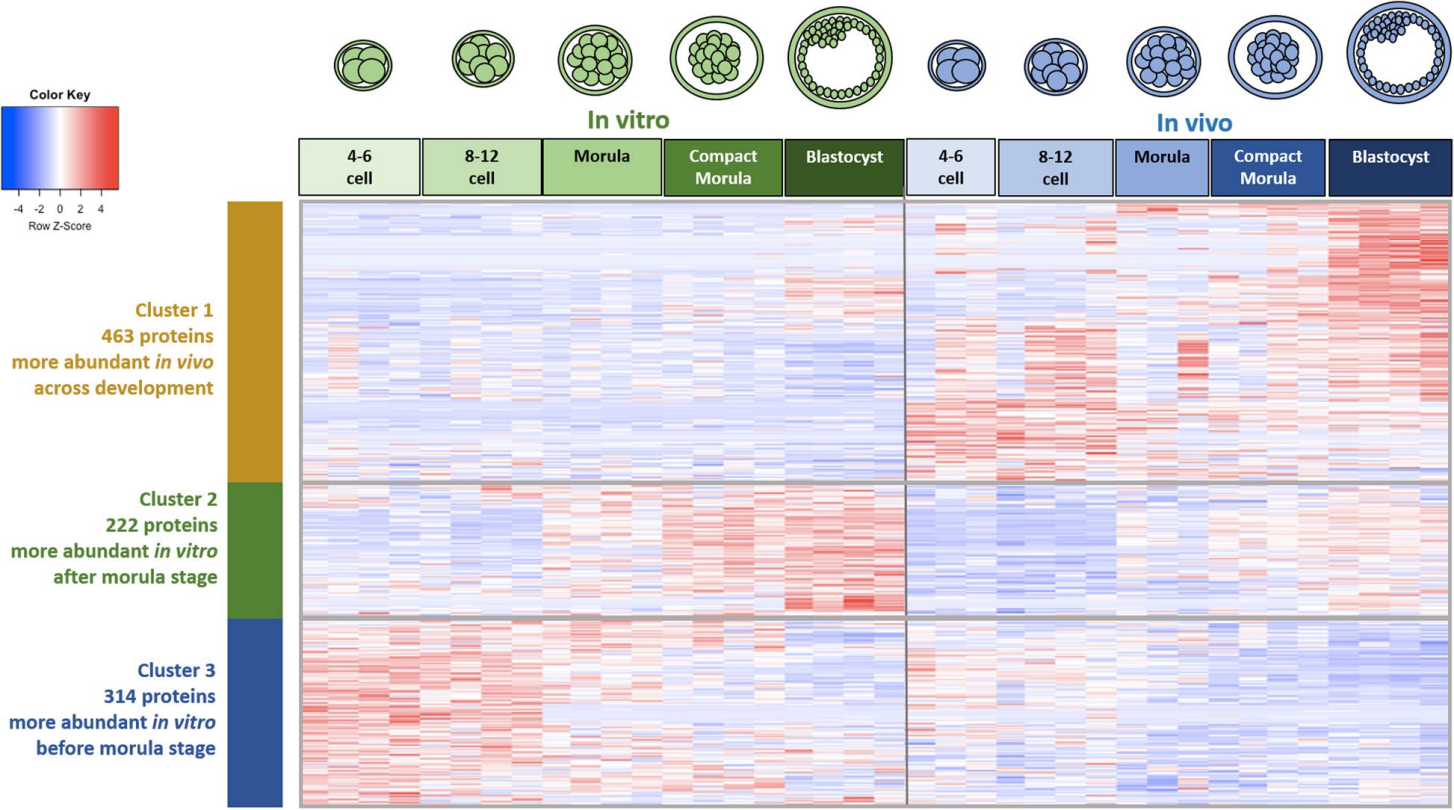
Heatmap and hierarchical clustering of differentially abundant proteins (DAPs) according to the origin of embryos. The 999 proteins with a *p*-value ≤ 0.050 after analysis of variance (ANOVA) were considered. Each line corresponds to one protein and each column to the normalized quantitative values of one embryo pool. The vertical grey line delimitates *in vitro*-derived embryos on the left from *in vivo*-derived embryos on the right. Red indicates higher abundance while blue indicates lower abundance compared with other conditions. Clusters of proteins identified after hierarchical clustering of data are delimited by colored vertical bars on the left and horizontal grey lines

The hierarchical clustering of differentially abundant proteins (DAPs) according to embryo origin allowed us to identify three clusters of DAPs illustrated in Fig. 3 (ANOVA *p*-value ≤ 0.050; see the list of DAPs in Table S2). Cluster 1 contained 463 proteins with higher abundance *in vivo* than *in vitro* across development. Cluster 2 contained 222 proteins that increased in abundance from the morula to blastocyst stages but displayed higher abundance in embryos developed *in vitro* compared to *in vivo*. Cluster 3 contained 314 proteins that decreased in abundance from the 4–6 cell to morula stages but displayed higher abundance *in vitro* than *in vivo*.

**Fig. 4:**
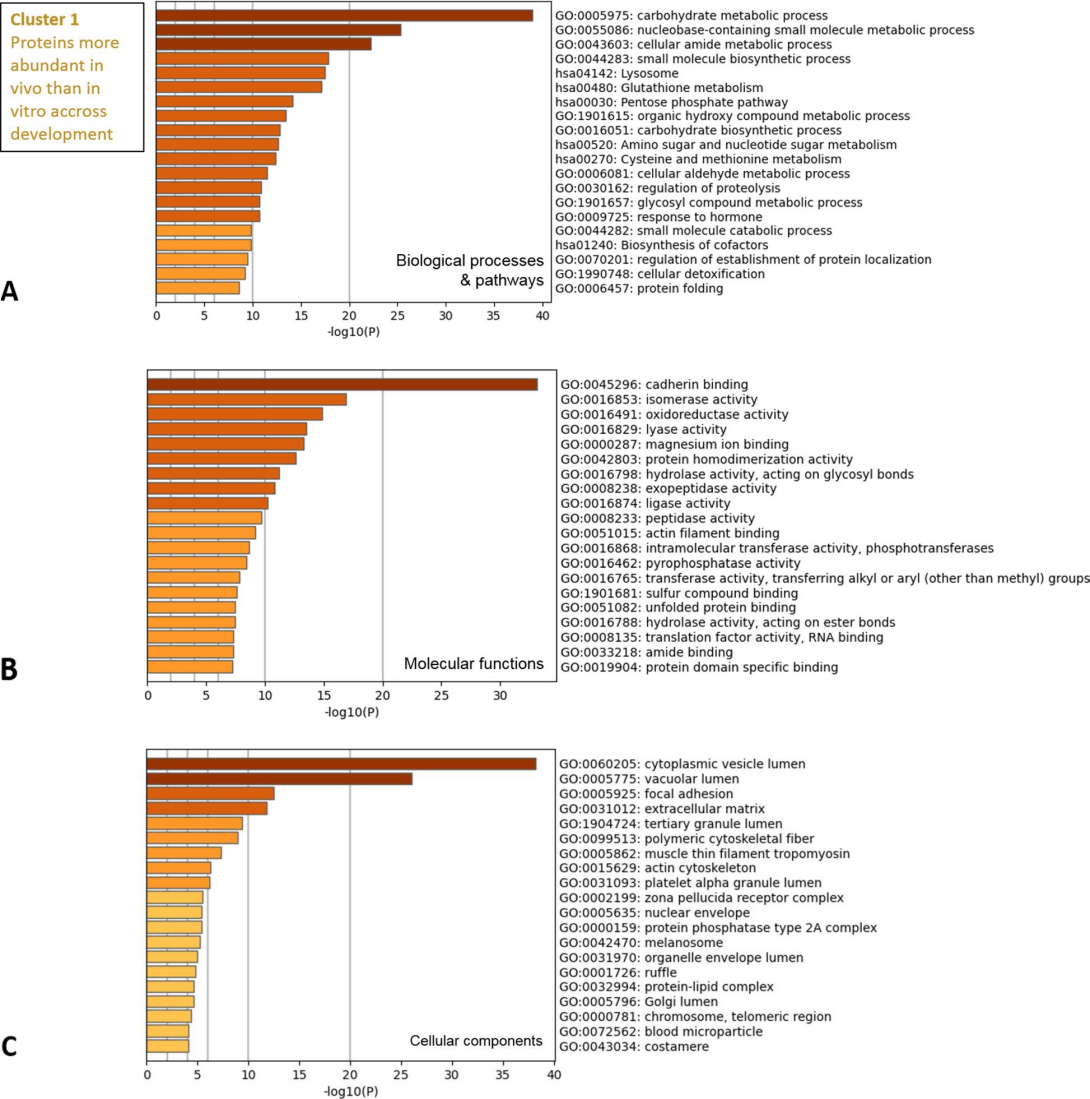
Functional enrichment analysis of proteins found more abundant *in vivo* than *in vitro* across development (cluster 1). The Metascape bar graphs represent the top 20 clusters of enriched gene ontology (GO) terms for biological processes and KEGG pathways (**A**), molecular functions (**B**), and cellular components (**C**). Each row represents one enriched cluster, and the darker color of the bars indicates higher significance (lower *p*-value). A -log10(P) of 20 corresponds to a *P*-value of 10^–20^. See Table S3 for the complete list of GO terms with corresponding gene names and *p*-values

The gene lists of the three clusters of DAPs were used for gene ontology (GO) enrichment analysis using the Metascape tool. The DAPs in cluster 1 were overrepresented in biological processes and pathways related to energy metabolism (carbohydrate metabolic and biosynthesis processes, small molecule biosynthetic process, pentose phosphate pathway, glycosyl compound metabolic process; see Fig. 4A and list of all enriched terms with related DAPs and p-values in Table S3). Another group of enriched processes and pathways was related to cellular detoxification (lysosome pathway, glutathione metabolism, regulation of proteolysis, cellular detoxification), while cadherin binding was the most enriched molecular function (Fig. 4B). The top enriched cellular components of cluster 1 included cytoplasmic components (cytoplasmic vesicle lumen, vacuolar lumen) and the extracellular compartment (focal adhesion, extracellular matrix) as the most significant terms (Fig. 4C and Table S3).

**Fig. 5:**
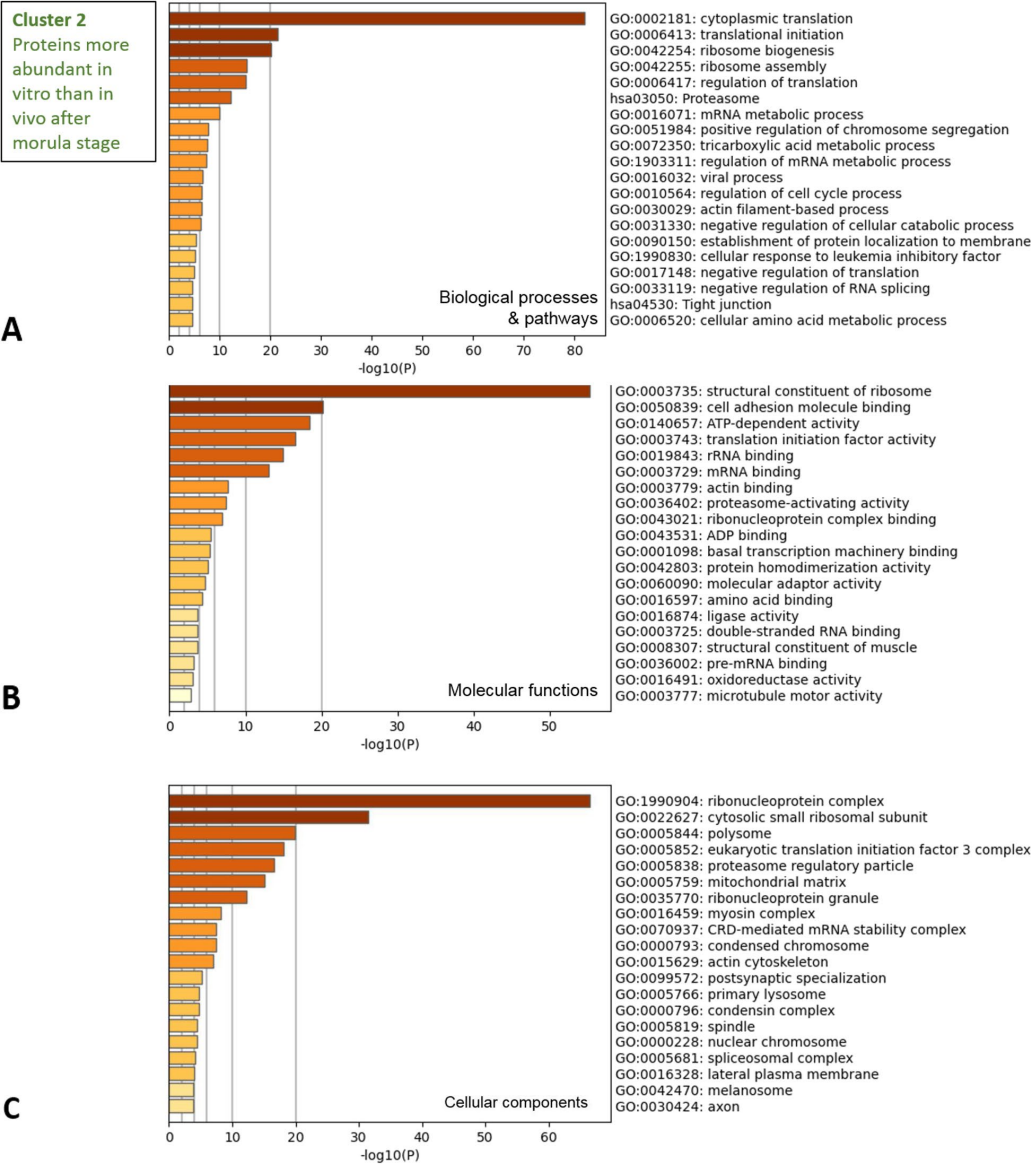
Functional analysis of proteins more abundant *in vitro* than *in vivo* after the morula stage (cluster 2). The Metascape bar graphs represent the top 20 clusters of enriched GO terms for biological processes and KEGG pathways (**A**), molecular functions (**B**), and cellular components (**C**). Each row represents one enriched cluster, and the darker color of the bars indicates higher significance (lower p-value). A -log10(P) of 20 corresponds to a *P*-value of 10^–20^. See Table S4 for the complete list of GO terms with corresponding gene names and *p*-values

The DAPs in cluster 2 were largely involved in protein synthesis; the most significant terms among the biological processes included cytoplasmic translation, translational initiation, ribosome biogenesis, ribosome assembly, and regulation of translation, among others (see Fig. 5A and Table S4 for details). In accordance, the enriched molecular functions were related to protein synthesis, including structural constituents of ribosomes, translation initiation factor activity, and ribonucleoprotein complex binding (Fig. 5B). The cell adhesion molecule binding was the second most enriched molecular function. The top cellular components of cluster 2 were involved in the translation machinery (ribonucleoprotein complex, cytosolic small ribosomal subunit, polysome, eukaryotic translation initiation factor 3 complex, among others; Fig. 5C).

**Fig. 6:**
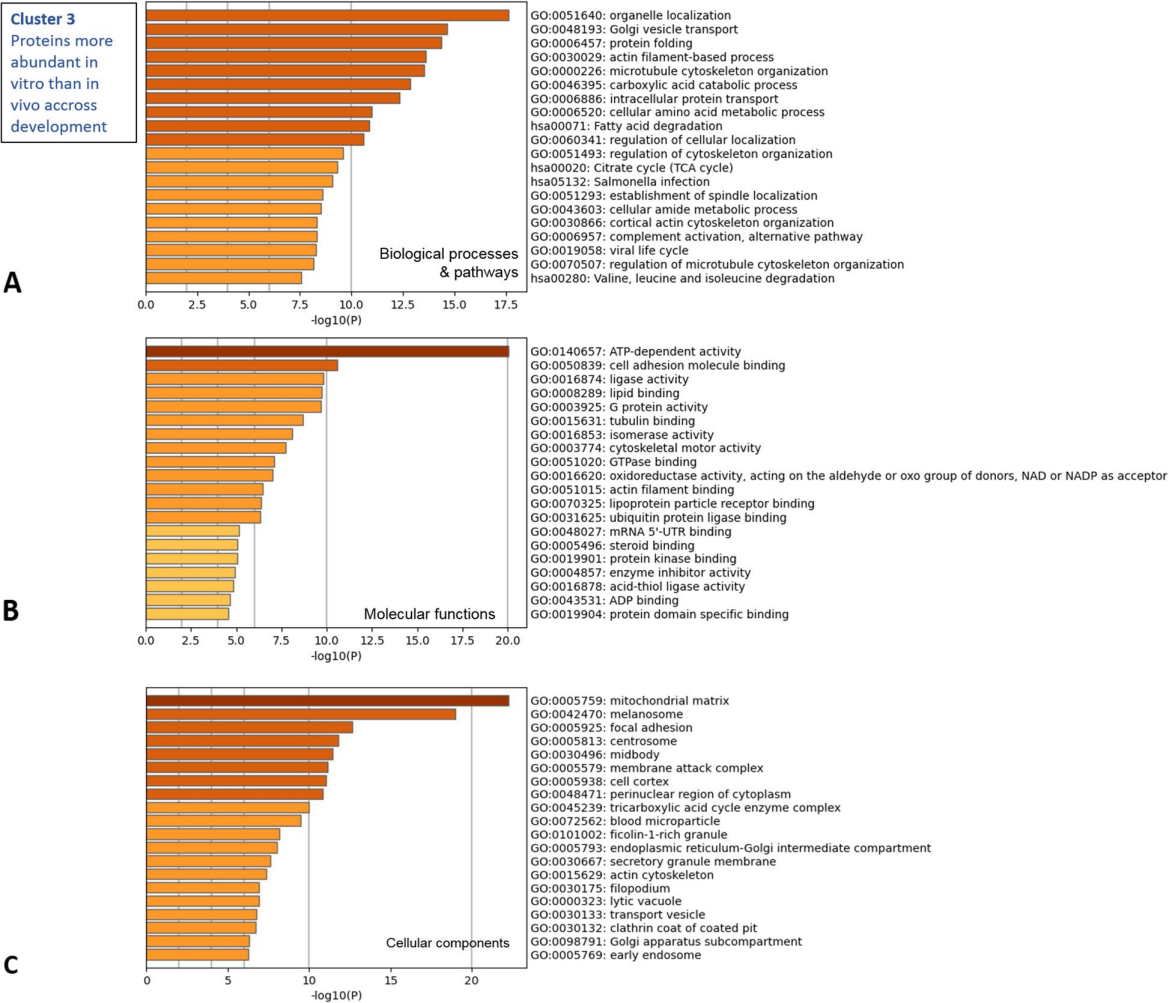
Functional analysis of proteins more abundant *in vitro* than *in vivo* before the morula stage (cluster 3). The Metascape bar graphs represent the top 20 clusters of enriched GO terms for biological processes and KEGG pathways (**A**), molecular functions (**B**), and cellular components (**C**). Each row represents one enriched cluster, and the darker color of the bars indicates higher significance (lower *p*-value). A -log10(P) of 10 corresponds to a *P*-value of 10^–10^. See Table S5 for the complete list of GO terms with corresponding gene names and *p*-values

The DAPs in cluster 3 were overrepresented in biological processes and pathways related to intracellular organization and protein processing, including organelle localization, Golgi vesicle transport, protein folding, actin filament-based processes, microtubule cytoskeleton organization, and intracellular protein transport (Fig. 6A; see details in Table S5). The most significantly enriched molecular functions were ATP-dependent activity followed by cell adhesion molecule binding (Fig. 6B). The most significantly enriched cellular component of cluster 3 was the mitochondrial matrix (Fig. 6C).

### Pairwise comparisons between *in vivo* and *in vitro*-derived embryos at each stage

**Fig. 7:**
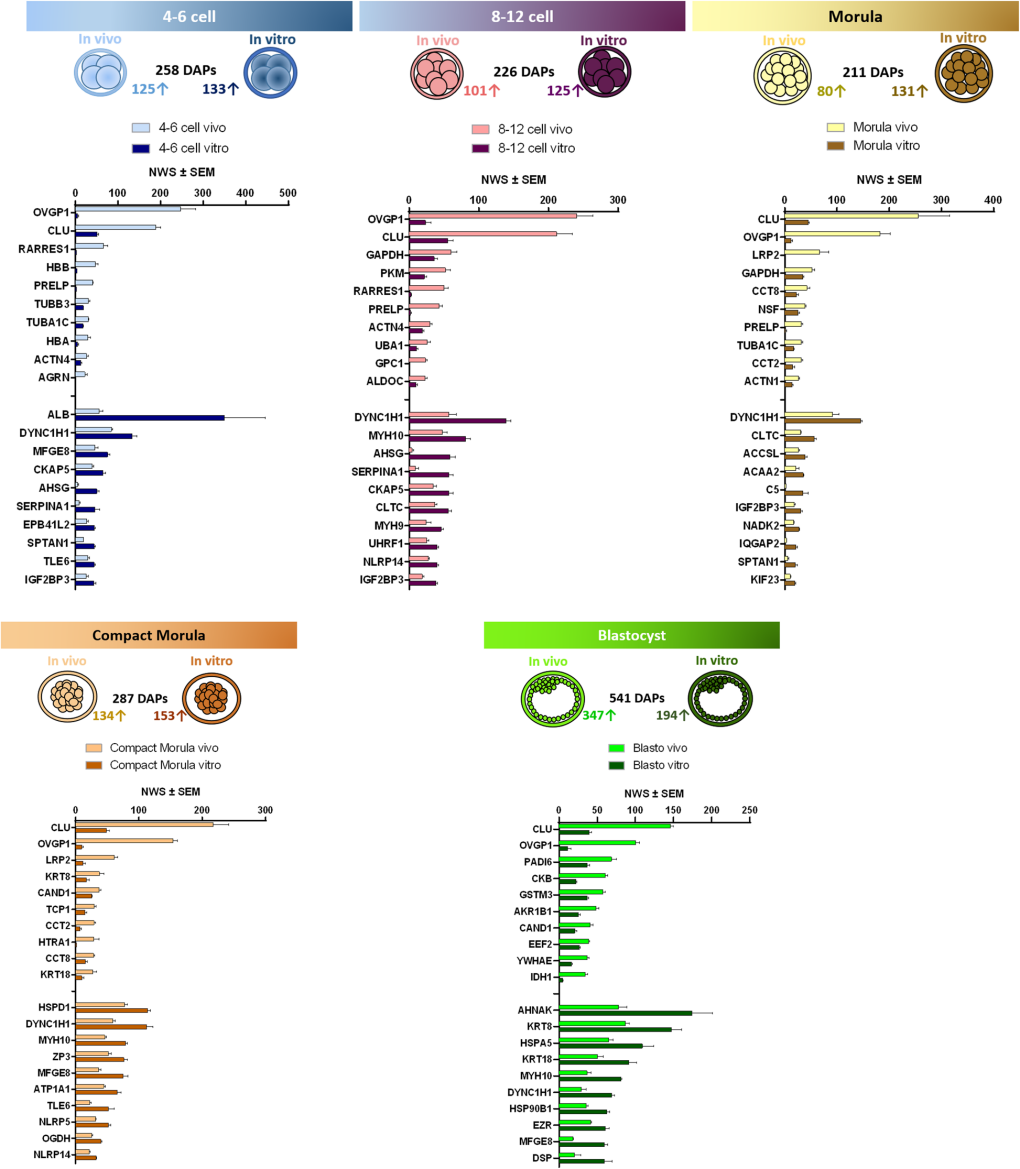
Top 20 DAPs after pairwise comparisons between *in vivo* and *in vitro*-derived embryos at each stage. Numbers of overabundant DAPs *in vivo* and *in vitro* (or less abundant DAPs *in vitro* and *in vivo*) are indicated beside the upward arrows. The histograms indicate the top 10 DAPs in each comparison. DAPs with a t-test *p*-value ≤ 0.05 and fold-change ratio ≥ 1.5 were considered

Pairwise comparisons between *in vivo* and *in vitro*-derived embryos retrieved between 211 and 541 DAPs at each stage (t-test *p*-value ≤ 0.05; fold-change ratios ≥ 1.5; overabundant protein ≥ 2 NWS). Figure 7 shows the total number of DAPs and those increased in abundance at each developmental stage and for each origin (see Table S6 for complete lists of DAPs with fold-change ratios and *p*-values).

**Fig. 8:**
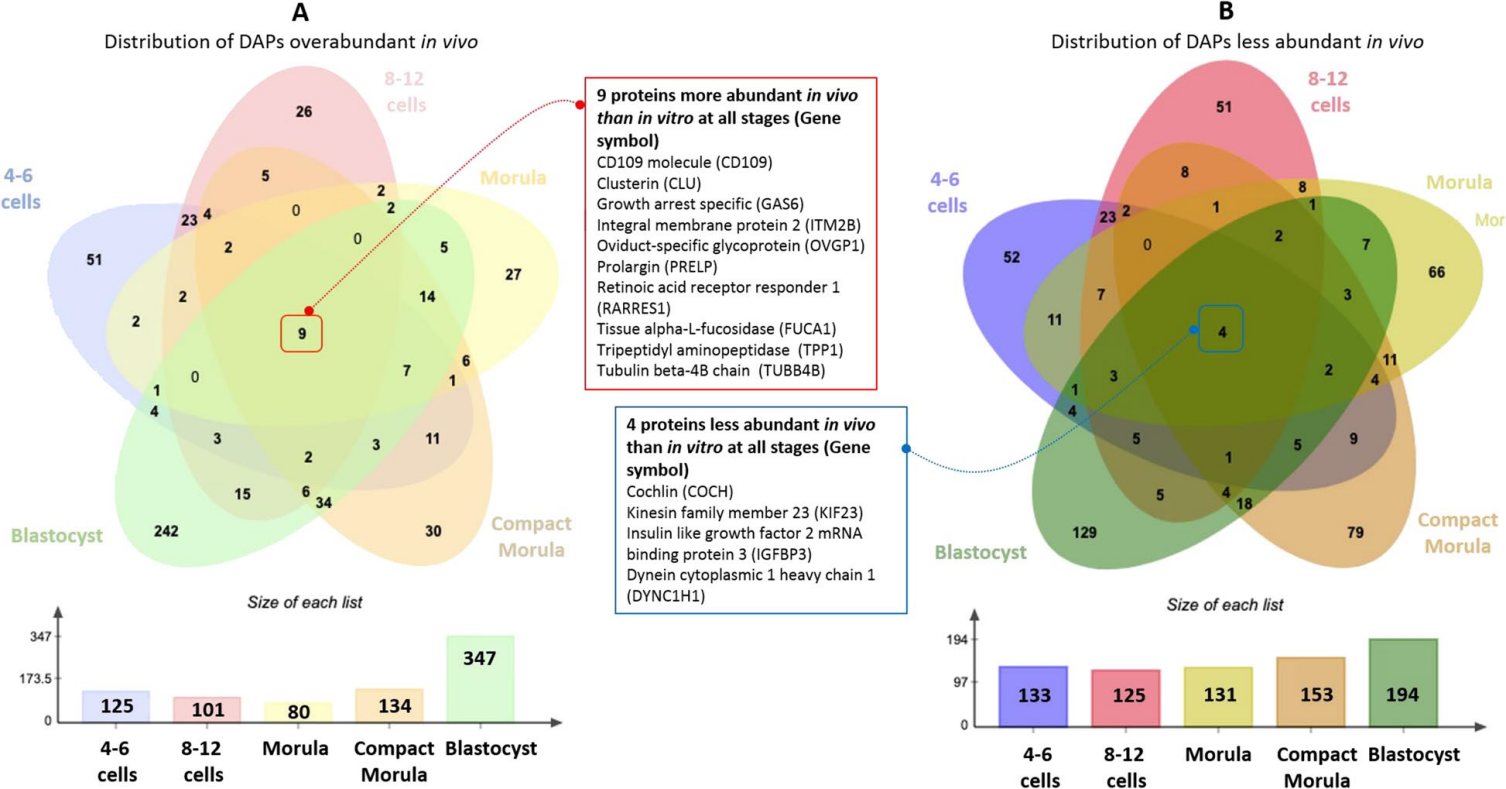
Distribution of proteins more (**A**) or less (**B**) abundant *in vivo* than *in vitro*. The Venn diagram indicates the overlap of DAPs between stages, and the histograms in the bottom indicate the number of DAPs at each stage. The frames indicate overabundant and less abundant DAPs, respectively, shared between all stages. DAPs with a t-test *p*-value ≤ 0.05 and fold-change ratio ≥ 1.5 were considered

The comparative analysis of DAPs across all stages evidenced nine proteins always more abundant *in vivo* than *in vitro*, including oviduct-specific glycoprotein 1 (also called oviductin, OVGP1), clusterin (CLU), CD109 molecule (CD109), retinoic acid receptor responder 1 (RARRES), and tissue alpha-L fucosidase (FUCA1; Fig. 8A). On the other hand, four proteins, including cochlin (COCH), kinesin family member 23 (KIF23), insulin like growth factor 2 mRNA binding protein 3 (IGF2BP3), and dynein cytoplasmic 1 heavy chain 1 (DYNC1H1), were always less abundant *in vivo* than *in vitro* (Fig. 8B; see Table S6 for the list of stage-specific DAPs after pair-wise comparisons).

**Fig. 9:**
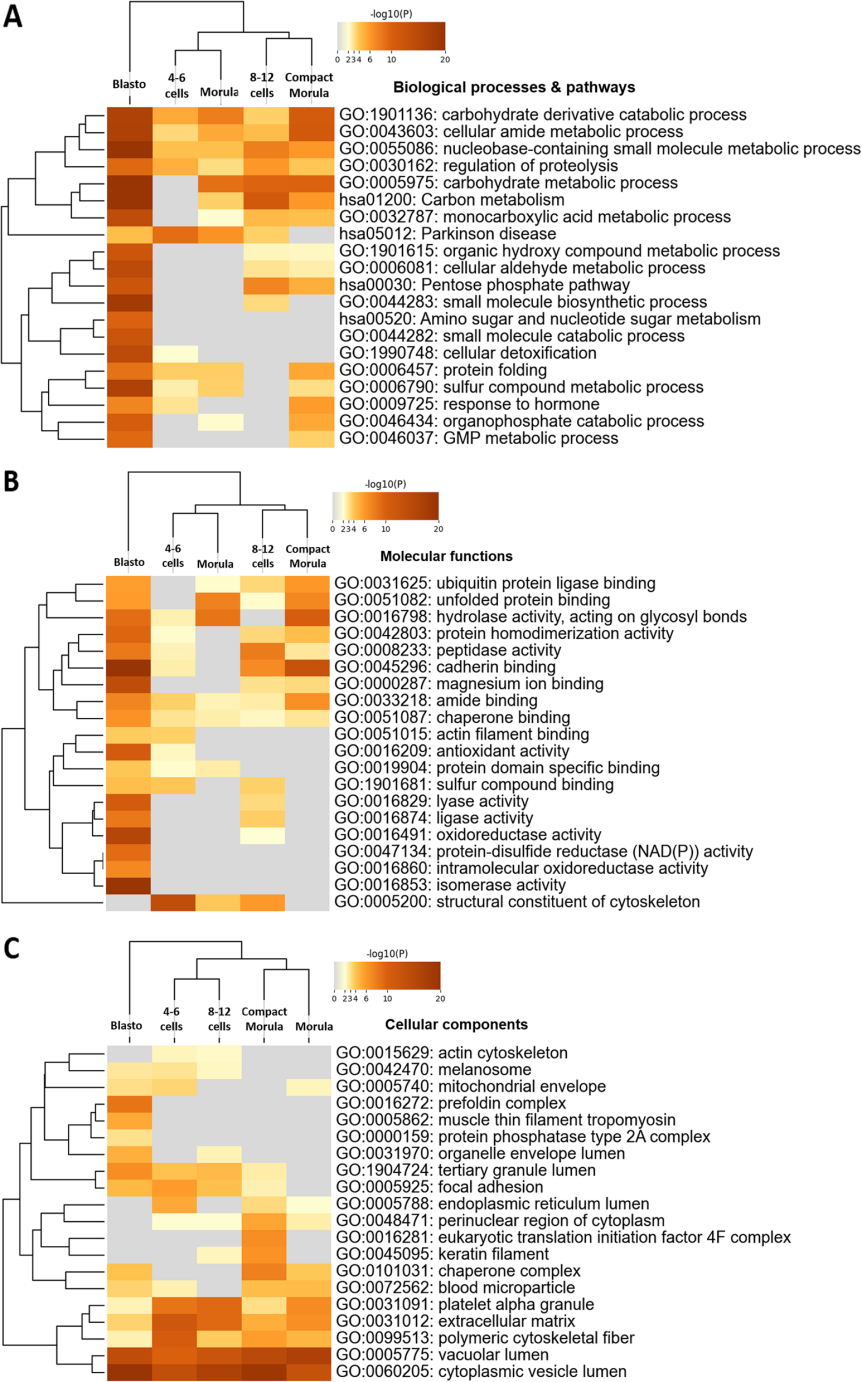
Functional analysis of proteins more abundant *in vivo* than *in vitro* at each developmental stage. The Metascape heatmap plots represent the top 20 clusters of enriched GO terms of biological processes and KEGG pathways (**A**), molecular functions (**B**), and cellular components (**C**). Each row represents one enriched cluster, and the orange gradation reflects statistical significance (the darker the color, the more significant the *p*-value is). Gray color indicates a lack of significance. DAPs with a t-test *p*-value ≤ 0.05 and fold-change ratio ≥ 1.5 were considered

The gene lists of DAPs in each *in vivo* vs. *in vivo* comparison were used for functional enrichment analysis using the Metascape tool. Figure 9A, B, and C show the significantly enriched GO terms of biological processes and pathways, molecular functions, and cellular components, respectively, for proteins more abundant *in vivo* than *in vitro* at each stage (see Table S7 for complete list of enriched terms). The most enriched processes/pathways were the carbohydrate derivative catabolic process and cellular amide metabolic process for all stages and carbohydrate metabolic process and carbon metabolism for all stages except the 4–6 cell stage. The blastocyst stage retrieved numerous highly significant processes, including small molecule catabolic processes and cellular detoxification. Enriched molecular functions at the blastocyst stage included cadherin binding, antioxidant activity, and intramolecular oxidoreductase activity. The most enriched cellular components for proteins found overabundant *in vivo* at all stages were the cytoplasmic vesicle lumen and vacuolar lumen.

**Fig. 10:**
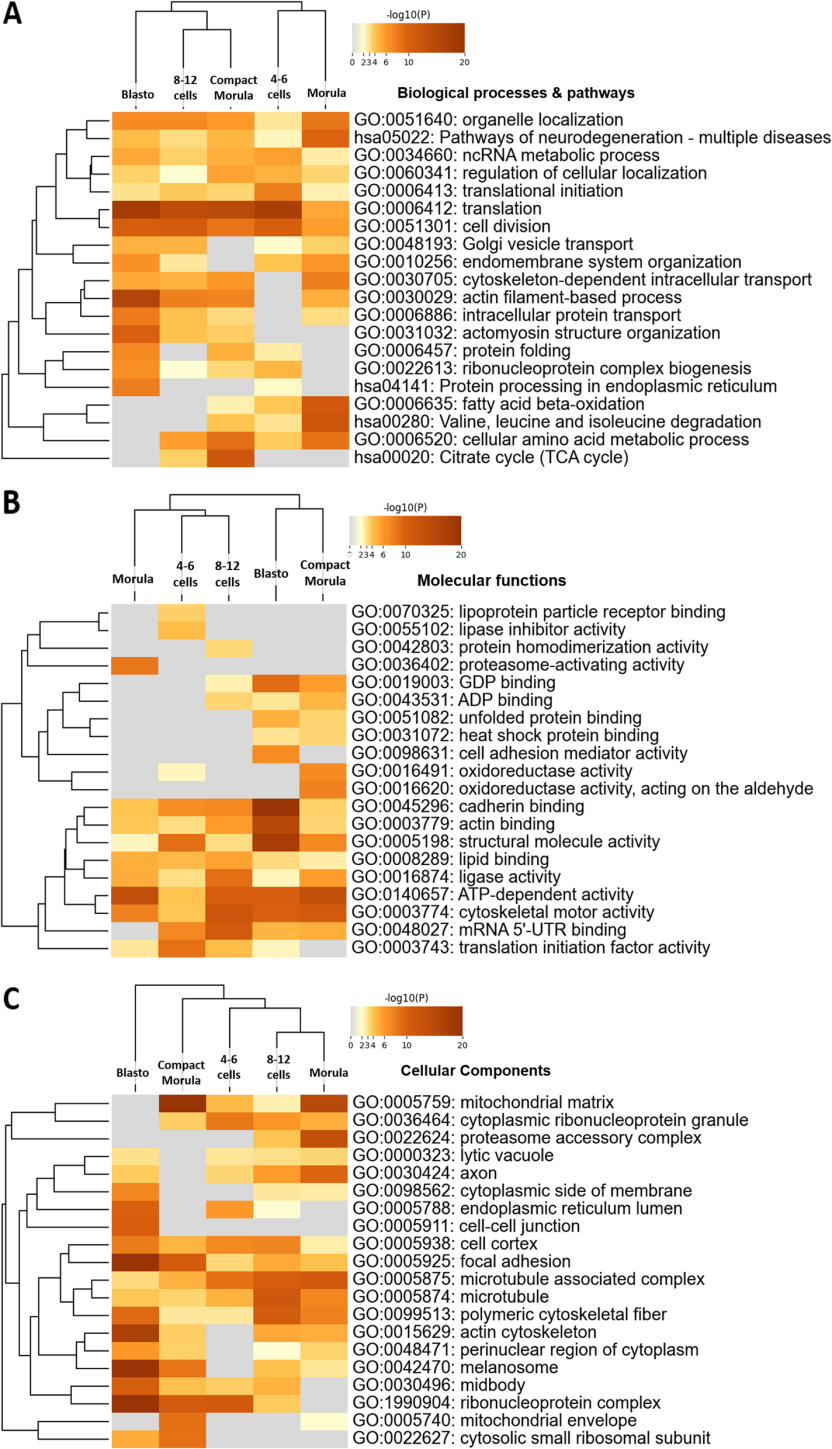
Functional analysis of proteins less abundant *in vivo* than *in vitro* at each developmental stage. The Metascape heatmap plots represent the top 20 clusters of enriched GO terms of biological processes and KEGG pathways (**A**), molecular functions (**B**), and cellular components (**C**). Each row represents one enriched cluster and the orange gradation reflects statistical significance (the darker the color, the more significant the *p*-value is). Gray color indicates a lack of significance. DAPs with a t-test *p*-value ≤ 0.05 and fold-change ratio ≥ 1.5 were considered

Figure 10A, B, and C show significantly enriched GO terms of biological processes/pathways, molecular functions, and cellular components, respectively, for proteins found less abundant *in vivo* than *in vitro* at each developmental stage (see Table S8 for complete list of enriched terms). Here, most enriched clusters were common to all stages. The most enriched processes/pathways were translation, cell division, organelle localization, and actin filament-based processes with high significance. Other biological processes included non-coding RNA metabolic process, translational initiation, and ribonucleoprotein complex biogenesis. The most enriched molecular functions for proteins found overabundant *in vitro* were ATP-dependent activity, cytoskeletal motor activity, and structural molecule activity. The most enriched cellular components were the mitochondrial matrix (all stages except blastocyst), ribonucleoprotein complex (all stages except morula), focal adhesion, and microtubule associated complex (all stages).

## Discussion

This study aimed at identifying the global contribution of the maternal environment to the embryo proteome and pointing out the alterations induced by *in vitro* embryo production. Previous studies have shown that the *in vivo* or *in vitro* maturation of oocytes is determinant for the developmental rate up to the blastocyst stage, while the culture environment of embryos is determinant for their quality in terms of gene expression, cryotolerance, and developmental ability [12, 20, 27–29]. The present data identify the global impact of *in vitro* oocyte maturation, fertilization, and culture conditions on the embryo proteome. To our knowledge, this is the first comparative proteomic analysis of preimplantation embryos developed either *in vivo* or *in vitro* in mammals. A total of 3,028 proteins were identified, representing the most comprehensive proteomic database in early embryos so far. Although the developmental stage was the first discriminating factor, the PCA of quantified proteins clearly differentiated embryos according to their origin as soon as the 4–6 cell stage. Overall, 46% of embryo proteins (999 of 2,184) were differentially abundant according to their origin, highlighting the great contribution of the maternal environment to the embryo proteome, in accordance with previous transcriptomic studies [17–21].

### Functional implication of proteins found less abundant *in vivo* than *in vitro* at early stages of development

Proteins in cluster 3 were more abundant in *in vitro*- than *in vivo*-derived embryos before the morula stage, as observed on the heatmap of DAPs. The mitochondrial matrix was the most significantly enriched cellular component in cluster 3 and included 38 proteins, including aconitate hydratase, mitochondrial (ACO2), fumarate hydratase, mitochondrial (FH), 3-ketoacyl-CoA thiolase, mitochondrial (ACAA2), ATP synthase subunit alpha, mitochondrial (ATP5F1A) and 2-oxoglutarate dehydrogenase, mitochondrial (OGDH) among the most abundant ones in 8–12 cell embryos produced *in vitro*. This indicates that although the cell numbers were the same, 4–6 cell and 8–12 cell embryos produced *in vitro* had higher abundance or lower degradation of mitochondrial proteins than their *in vivo* counterparts. Mitochondria are maternally inherited in embryos and do not replicate until embryonic differentiation at the morula stage, leading to a progressive decrease of mitochondrial copies per blastomere at each cell division [30]. In accordance, ATP-dependent activity, a mitochondrial activity, was the most enriched molecular function for proteins in cluster 3 and also significantly enriched in proteins found overabundant *in vitro* at all stages. The mitochondrial content of bovine oocytes lacks data; however, in humans, the number and ultrastructure of mitochondria do not differ between *in vivo*- and *in vitro*-matured oocytes [31]. Furthermore, the general ATP content per oocyte did not differ according to *in vivo* vs. *in vitro* origin in pigs [32]. Thus, it is likely that bovine *in vitro*-derived embryos had initially similar content in mitochondrial proteins but slower decrease in these proteins over time. These results suggest that *in vitro* culture conditions impaired the capacity of embryos to properly use the oocyte-derived mitochondrial proteins. Consistent with this hypothesis, previous studies on bovine blastocysts observed a larger amount of mitochondrial debris [10] and DNA copy numbers [33] in *in vitro-* compared to *in vivo*-derived embryos. ATP is an important energy source for protein synthesis and all cellular functions. Further studies would be necessary to see if this slower decrease in mitochondrial proteins *in vitro* is associated with dysregulation of ATP synthesis.

Other proteins in cluster 3 may be the result of higher initial abundance and/or slower use of oocyte-derived proteins in *in vitro*-derived embryos. For instance, metalloendopeptidase (ASTL), cochlin (COCH), enoyl-CoA delta isomerase 2 (ECI2), endoplasmic reticulum resident protein 44 (ERP44), SLC3A2 protein (SLC3A2), Tudor and KH domain containing protein (TDRKH) and the uncharacterized protein LSM14B were all included in cluster 3 and previously identified in a cluster of proteins that gradually decreased in abundance from metaphase II oocytes to 4-cell embryos in cattle [34].

Proteins in cluster 3 were overrepresented in pathways related to intracytoplasmic organelle localization and actin filament-based processes. In accordance, at early stages of development, proteins overabundant *in vitro* were involved in cell division, actin filament-based process, cytoskeletal motor activity, and structural molecule activity as the most enriched pathways and functions. These pathways included numerous proteins including annexins (ANXA11), myosins (MYO1B, MYH9, MYH10, MYH11, MYO1C…), and spectrins (SPTAN1, SPTBN1). This indicates that pathways related to cellular organization and cell division were dysregulated in *in vitro*-derived embryos, which could impair embryo morphology and kinetics of development. Morphological evaluation is widely used to evaluate embryo quality, and only embryos of grade-1 quality, i.e., consistent with their expected stage of development, with uniform blastomeres and few irregularities, were used for the present study. Furthermore, although the timing of fertilization is impossible to determine *in vivo*, the time of embryo collection after insemination was similar to the time of embryo collection after *in vitro* fertilization (Table 1), indicating globally similar rates of development. Although their morphology was similar to those developed *in vivo*, some embryos developed *in vitro* tended to appear darker than *in vivo*-developed embryos, probably due to the accumulation of lipid droplets and increased debris in the perivtelline space and intercellular cavities [9–12, 17]. Therefore, proteomic differences may in part explain the reported ultrastructural alterations in bovine *in vitro*-produced embryos, which are associated with lower tolerance to cryopreservation and developmental ability.

**Table 1:** Pools of *in vivo*- and *in vitro*-derived bovine embryos used for nanoLC-MS/MS analysis (*n* = 4 embryos per pool)

**Embryonic stage**	**Origin**	**Time of embryo collection after AI1 or IVF**^a^	**Female donors**^b^	**Bull semen**^c^	**Number of pools**
**4–6 cells**	*In vivo*	Days 1.7–3.8	A, B, C, D	X	3
	*In vitro*	Day 2		X	4
**8–12 cells**	*In vivo*	Days 3.6–3.8	A, B	X	4
	*In vitro*	Day 3		X	4
**Morula (> 16 cells)**	*In vivo*	Days 5.7–6.7	E, F, G	X	3
	*In vitro*	Days 5–6		X	4
**Compact morula**	*In vivo*	Days 5.7–6.8	E, F, H	X	4
	*In vitro*	Day 6		X	4
**Blastocyst**	*In vivo*	Days 6.8–7.5	H, I, J, K	X, Y, Z	4
	*In vitro*	Days 7–8		X	4

^a^*IA1* first artificial insemination of donor cows for *in vivo*-derived embryos, *IVF* *in vitro* fertilization

^b^A total of 9 Holstein cows (called A to I) and 2 Holstein heifers (called J and K) were used as female donors for *in vivo*-derived embryos. *In vitro*-derived embryos were produced from ovaries collected at a commercial slaughterhouse

^c^A total of 3 bulls of proven fertility, one Normande (called X) and two Holstein (called Y and Z), were used for the production of *in vivo*-derived embryos; the X bull was used for *in vitro*-produced embryos

### Differences in embryo genome activation between *in vivo*- and *in vitro*-derived embryos 

The switch from mRNA and proteins derived from the oocyte to those resulting from embryonic genome activation (EGA) is a critical step during embryo development. Based on previous transcriptomic data, the onset of EGA in cattle was timed between the 8-cell and 16-cell stages [8, 35–37]. Our previous proteomic analysis of *in vivo*-derived embryos evidenced a significant increase in proteins involved in protein synthesis between the 8–12 cell and morula stages, consistent with a major EGA starting around the 8-cell stage [26]. The present results confirm that EGA occurred approximately at the same time in *in vitro*-derived embryos; a marked increase in the abundance of proteins involved in protein synthesis was clearly seen after the 8–12 cell stage, resulting in increased numbers of proteins identified per embryo up to the blastocyst stage. This is in line with previous data that showed an increase in the uptake of ^35^S-methionine to measure de novo protein synthesis in embryos starting from the 8- to 16-cell stage in both *in vivo*- and *in vitro*-derived bovine embryos [22].

However, dynamics in total numbers of proteins identified slightly differed between *in vivo* and *in vitro*-derived embryos around EGA. While protein numbers reached a nadir in *in vivo*-derived embryos at the morula stage then increased again up to the blastocyst stage, *in vitro*-derived embryos had constantly increased numbers of proteins. Furthermore, the increase in proteins involved in protein synthesis was much stronger and faster in *in vitro*-derived embryos compared to their *in vivo* counterparts, resulting in the identification of cluster 2 regrouping proteins largely involved in protein synthesis.

The most significant enriched GO terms in cluster 2 were related to cytoplasmic translation and included multiple ribosomal proteins (of the small 40S and large 60S ribosomal subunits, RPSs and RPLs, respectively) as well as eukaryotic translation initiation factors (EIF4G1, EIF4G2, EIF3E…) and proteins involved in positive regulation of protein translation (DDX3X, HNRNPU, HSPB1…). In accordance, translation and other biological processes related to RNA metabolism were among the most significantly enriched pathways for proteins found more abundant *in vitro* at each stage. In accordance with our results, the uptake of ^35^S-methionine was significantly higher in *in vitro*-produced bovine embryos as compared to *in vivo*-derived embryos from the 8–16 cell to the blastocyst stages [22]. A culture effect on the expression of genes involved in gene expression was also observed by comparing the transcriptomic profiles of bovine blastocysts fertilized *in vitro* or *in vivo* [19].

The present results indicate that *in vitro* fertilization and culture conditions led to a higher abundance of proteins involved in RNA metabolism and translation at the time of EGA. Although spending more resources for protein synthesis, the blastocysts produced *in vitro* did not display a higher number of proteins compared to *in vivo*-developed blastocysts, in line with previous data reporting similar total protein contents in bovine blastocysts of both origins [38]. The higher abundance of translation-related proteins in *in vitro*-derived embryos may be due to an upregulation of genes coding for those proteins coupled or not with a lower turn-over of those proteins, as compared to *in vivo*. These results are globally in accordance with the “quiet embryo” hypothesis, in which a premature or an excessive activation of the embryonic genome, in response to an unfavorable environment, decreases the ability of an embryo to pursue development [22]. In the “quiet embryo” hypothesis, it was suggested that the embryos possessing a greater ability to recycle endogenous proteins via specific degradation pathways before EGA has an advantage to pursue development [22]. We speculate that the upregulation of proteins involved in protein synthesis in embryos produced *in vitro* may compensate for their lower consumption of oocyte-derived proteins before EGA.

### Specificity of the blastocyst stage

The blastocyst stage retrieved several particularities in the present study. The number of proteins identified were highest in both groups of blastocysts compared with earlier stages. This was expected as protein synthesis has been reported to overcome protein degradation after EGA, leading to an increased amount of proteins per embryo [38]. The PCA of all quantified proteins evidenced gaps between blastocysts and compact morulas in both groups of embryos and also between *in vivo* and *in vitro* blastocysts. This was confirmed after pair-wise comparison of proteins at each stage, which retrieved the highest number of DAPs in blastocysts. In accordance, the functional analysis of DAPs at the blastocyst stage evidenced several processes and pathways overrepresented only at this stage.

In order to decrease genetic heterogeneity between embryos, only one Normande bull was used for the production of all embryos produced *in vitro* and most embryos produced *in vivo*. While all embryos developed *in vivo* up to the compact morula stage had the same bull as *in vitro*-fertilized embryos as progenitor, 10 out of 16 *in vivo*-derived blastocysts were obtained with two additional Holstein bulls. This was needed for technical, economical, and ethical reasons as donor cows did not provide enough blastocysts. As a result, *in vivo* blastocysts originated from three fathers, whereas all other embryos had one father out of the three previous ones. However, the three bulls used had very similar fertility, as assessed on a large population of cows and heifers (59%–62% and 45%–49% of non-return rates at 90 days after insemination on more than 3,200 and 22,800 heifers and cows, respectively). It has been shown that the father has an impact on gene expression and epigenomics in bovine blastocysts [39, 40], which may account for differences in protein abundance between blastocysts in this study.

### Higher abundance of proteins involved in carbohydrate metabolism in *in vivo*-derived embryos

The most crucial nutrients for early developing embryos are carbohydrates and amino acids, which not only provide energy and substrates for protein synthesis but act as players of epigenetic programming [41]. The DAPs found more abundant in the *in vivo*-derived embryos compared to *in vitro*-derived embryos were largely involved in energy metabolism. For proteins overabundant *in vivo* across development (cluster 1) and at each stage (after pair-wise comparisons), the most significantly enriched GO terms were related to carbohydrate metabolism. In accordance, previous transcriptomic studies evidenced that the most significant GO terms of differentially expressed genes between bovine *in vivo*- and *in vitro*-derived blastocysts were related to metabolic processes, including carbohydrate metabolism but also lipid, nucleic acid, and amino acid metabolism [17, 18]. Similarly, a high proportion of genes related to metabolism was dysregulated when porcine *in vivo*- or *in vitro*-derived blastocysts were compared, most of which were upregulated *in vivo* [42].

When considering *in vivo*-derived embryos, the enrichment in the carbohydrate metabolic and catabolic processes started at the 8–12 cell stage and reached highest significance at the compact morula and blastocyst stages. Our results indicate that *in vivo*-derived embryos allocated more resources toward the synthesis of key glycolytic enzymes compared to their *in vitro* counterparts as soon as the 8–12 cell stage. Around this stage, bovine embryos enter the uterus, where concentrations of glucose are higher than in the oviduct fluid [43]. Our results are in accordance with previous reports on bovine embryos that observed the first increase in pyruvate and glucose utilization between the 8- and 16-cell stages, i.e., around the time of EGA, and significant increases in the oxidation of pyruvate, glucose, and lactose at the time of morula compaction [14, 15]. The proteins found more abundant in the *in vivo*-derived embryos of this study regrouped proteins of the pyruvate metabolic pathway (fructose bisphosphate aldolase, ALDOA; phosphoglycerate mutase, BPGM; galactokinase, GALK1; L-lactate dehydrogenase A and B chains, LDHA and LDHB; phosphoglycerate mutase 1, PGAM1; phosphoglycerate kinase 1, PGK1; phosphoglucomutase-1, PGM1…) as well as many proteins involved in the glycolytic (GAPDH, GPI, HK2, PGK1, PGM1 and alpha-enolase, ENO1) and pentose phosphate (glucose-6-phosphate 1-dehydrogenase, G6PD; 6-phosphogluconate dehydrogenase, decarboxylating, PGD; ribose-5-phosphate isomerase, RPIA; transketolase, TKT; transaldolase, TALDO1) pathways. It is well established that early mammalian embryos use mostly pyruvate as the preferred carbohydrate, which is metabolized through the tricarboxylic acid (TCA) cycle and oxidative phosphorylation to produce ATP, then when the demand for energy increases, glucose is metabolized through the pentose phosphate pathway and the glycolysis pathway, in addition to the TCA cycle [44].

While the large majority of studies on embryo metabolism used *in vitro*-derived embryos, there are few comparing carbohydrate metabolism of *in vivo*- and *in vitro*-derived embryos at the same stages of development and within the same experiments [14, 45]. Globally similar patterns of glucose, pyruvate, and lactate consumption were observed in bovine early embryos of both origins, except a lower carbon uptake and higher rate of lactate conversion from glucose (i.e. higher glycolysis) after the 16-cell stage in *in vitro*-derived embryos as compared to their *in vivo* counterparts [14]. In the present study, most enzymes involved in glucose-to-lactate conversion were found more abundant in *in vivo*-derived embryos after the 8–12 cell stage (hexokinase HK2; glyceraldehyde-3-phosphate dehydrogenase, GAPDH; phosphoglycerate kinase 1, PGK1) or at all stages (glucose-6-phosphate isomerase, GPI; phosphoglucomutase-1, PGM1; alpha-enolase, ENO1; L-lactate dehydrogenase, LDHA). This is suggestive of a higher glycolytic rate in *in vivo*-derived embryos. Proteins of the pentose phosphate pathway were also more abundant in *in vivo*-derived embryos, suggesting that glucose may also be converted into NADPH and pentoses at a higher rate as compared to *in vitro*-derived embryos.

Although *in vivo*-derived embryos had low abundance of proteins dedicated to protein synthesis suggestive of a “quiet EGA”, their carbohydrate metabolism seemed more active as compared to *in vitro*-produced embryos. According to our results, the allocation of resources toward carbohydrate metabolism rather than protein synthesis at the time of EGA may be predictive of higher developmental ability. This does not support the hypothesis of the “quiet metabolism”, that is a low rather than active metabolism could support better embryo viability and further development [22, 23]. Interestingly, intermediate, but not low, levels of pyruvate consumption have been identified to be predictive of bovine embryo quality and the ability to develop up to the blastocyst stage *in vitro* [24]. Although the “quiet embryo” hypothesis has been recently reworked to propose an optimal range of metabolic activity depending on individual embryos [46], our results are more in favor of an active carbohydrate metabolism to pursue development.

### Oviduct origin of proteins found more abundant in *in vivo*-derived embryos

Overabundant proteins in *in vivo*-derived embryos may result from higher protein synthesis, lower protein degradation, and/or internalization from the maternal tract fluids. As discussed above, higher protein synthesis in *in vivo*-derived embryos is unlikely, especially after EGA, as there was no increase in the total amount of proteins identified. Furthermore, contrary to cluster 2, proteins in cluster 1 were not functionally implied in protein synthesis. It is probable that some proteins present in the post-ovulatory oviduct fluid and able to cross the zona pellucida were internalized by *in vivo*-derived embryos. It is to note that *in vivo*-derived embryos were extensively washed before analysis to avoid contamination with oviduct/uterine fluids at the surface of the zona pellucida.

Many studies using various culture conditions mimicking the maternal environment have shown that the secretions of the oviduct are determinant for early embryo quality [28, 47–49]. However, very few oviductal proteins involved in embryo development have been identified to date, and most studies used *in vitro*-derived embryos [25, 50–53], which gives a biased picture of this issue. It has been well established that OVGP1, a glycoprotein of around 100 kDa secreted in the oviduct fluid, has the ability to cross the zona pellucida and be internalized by developing mammalian embryos [25, 54–57]. From *in vitro* studies, positive effects of purified or recombinant OVGP1 on embryo development have been evidenced in mice [53], goats [52], and cattle [51]. It appears from our data that OVGP1 is not strictly speaking an oviduct-specific protein since OVGP1 was detected in all *in vitro*-developed embryos (at 6 to 23 NWS on average), in accord with a previous study that detected low levels of OVGP1 mRNAs in bovine 8-cell embryos produced *in vitro* without oviduct cells or fluid [58]. Nevertheless, OVGP1 was on average 9- to 44-fold more abundant in embryos developed *in vivo* as compared to those produced *in vitro* (mean fold-change ratios of 44, 10, 16, 16, and 9 at the 4–6 cell, 8–12 cell, morula, compact morula, and blastocyst stages, respectively), confirming that this protein is internalized by embryos from the start of development. In accordance, previous observation using immunolabelling of OVGP1 in embryos collected *in vivo* revealed that this protein localized in the perivitelline space and inside embryo blastomeres in baboons [55], golden hamsters [56], pigs [57], and cattle [25, 54].

Some other proteins found overabundant in the *in vivo*-derived embryos were previously identified as interacting with *in vitro*-produced embryos after incubation with post-ovulatory oviduct fluid, including retinal dehydrogenase 1 (ALDH1A1) and alpha isoform of regulatory subunit A protein phosphatase 2 (PPP2R1A) at the 4–6 cell stage, cytoplasmic aconitate hydratase (ACO1), hemoglobin subunit beta (HBB) and purine nucleoside phosphorylase (PNP) at the morula stage, and annexin A8 (ANXA8), creatine kinase B-type (CKB), chloride intracellular channel protein 1 (CLIC1), cytosolic non-specific dipeptidase (CNDP2), hemoglobin subunit alpha (HBA), ribonuclease inhibitor (RNH1), tubulin polymerization-promoting protein family member 3 (TPPP3), and CD109 at both stages [25]. A comparison between proteins in cluster 1 and those identified by MS in the isthmic part of the oviduct fluid just after ovulation [59], i.e., place and time of early embryo development, indicates that 341 (75%) are also present in the oviduct fluid (data not shown). Among those, OVGP1, CD109, CLU, RARRES1, and tubulin beta-4B chain (TUBB4B) were quantified at high abundance in the postovulatory oviduct fluid (36 to 121 NWS, [59]) and were found overabundant in *in vivo*-derived embryos from the 4–6 cell up to blastocyst stages, making them good candidates for embryo-interacting proteins originated in the oviduct.

The present database is valuable for further functional studies on embryo-maternal interactions and may allow us to exclude some hypotheses, at least in the bovine species. For instance, complement C3 was previously reported as an oviductal cell-derived protein with embryotropic activity in rodents [60, 61] and shown to be internalized by rat embryos just after implantation [61]. In this study, C3 was more abundant *in vitro* than *in vivo* from the 4–6 cell to compact morula stages and identified in cluster 3. Therefore, although C3 was reported at high abundance in the bovine post-ovulatory OF [59], the present data do not support any internalization by cattle embryos *in vivo*.

### Limitations of the study

For technical and ethical reasons, female donors were synchronized for estrus and treated for superovulation, which may modify the endocrine environment of *in vivo*-developed embryos compared to classical insemination [62] and have consequences on their proteome. In addition, the number of bulls used as progenitors was voluntarily low, which probably helped identify differences between *in vivo* and *in vitro*-derived embryos by decreasing genetic heterogeneity but does not reflect the proteomic diversity of bovine embryos.

*In vitro*-produced embryos were cultured without any protein or serum supplementation to avoid protein contaminant. The absence of proteins in the culture medium may have induced specific pathways compared with embryos developed in the presence of serum or proteins in the culture medium.

## Conclusions

The maternal environment led to higher degradation of mitochondrial proteins at early developmental stages, lower abundance of proteins involved in protein synthesis at the time of embryonic genome activation, and a global upregulation of carbohydrate and small molecule metabolic pathways compared to *in vitro* production. Furthermore, our data confirm that embryos developed *in vivo* uptake large amounts of OVGP1 and probably other oviduct fluid-derived proteins as soon as the 4–6 cell stage. These data provide new insight into the molecular contribution of the maternal environment to the developmental ability of early embryos. Moreover, the DAPs identified constitute valuable markers of embryo quality and target pathways for the assessment of new *in vitro* systems, closer to *in vivo* conditions.

## Methods

### Collection of embryos developed *in vivo* from synchronized donors

The protocol used for embryo collection was reported previously [26]. A total of 11 Holstein females (9 dried off cows and 2 heifers, aged 1–5 years) housed at the INRAE Experimental Unit of Animal Physiology of the Orfrasière (Nouzilly, France) were used as donor animals. The estrus cycles of the donors were synchronized with the prostaglandin analog cloprostenol (0.5 mg, i.m.; Estrumate, MSD Animal Health, Beaucouzé, France) and all females showing normal estrus and an ovarian corpus luteum 7 days after estrus (checked by ultrasonography) received a progesterone-releasing intravaginal device (PRID DELTA, Ceva Santé animale, Libourne, France). Ovarian stimulation treatment started 12 days after estrus and consisted of eight intramuscular injections of decreasing porcine Follicle stimulating hormone (pFSH)/porcine Luteinizing hormone (pLH) doses every 12 h over 4 days (Stimufol, Reprobiol, Liège, Belgique; 500 µg pFSH and 100 µg pLH in total in cows; 350 µg pFSH and 70 µg pLH in total in heifers). Luteolysis was induced with 2 mL of cloprostenol (0.5 mg, i.m; Estrumate, MSD Animal Health, Beaucouzé, France) and the PRID DELTA device was withdrawn together with the fourth and sixth FSH administrations, respectively.

For embryo collection, donor females were inseminated twice with frozen-thawed semen from one bull of proven fertility 12 and 24 h after standing estrus. The same ejaculate from one Normande bull (called X) was used for all embryos except 10 blastocysts for which two extra Holstein bulls (called Y and Z) were used (Table 1). Bulls’ fertility was previously estimated by the 90-day non-return rate after artificial insemination (AI) on a large population of dairy females and ranged from 59 to 62% in heifers (3,241–8,587 AI per bull) and from 45 to 49% in lactating cows (22,864–26,623 AI per bull). The 10 blastocysts from bulls Y and Z were collected by non-invasive cervical flushing of the uterine horns of heifers (one flushing session per heifer) on day 7 after the first AI, as previously described [26]. All other *in vivo*-derived embryos were recovered after the slaughter of donor cows between days 1.7 and 7.5 after the first AI in the local experimental slaughterhouse. The genital tracts were immediately processed on site. Oviducts and uterine horns were flushed with 5 mL and 35 mL, respectively, of prewarmed phosphate buffered saline (PBS) and 0.1% polyvinyl alcohol (PVA) at 38.5 °C. Recovered fluids were carefully observed for the presence of embryos under a stereomicroscope, and all embryos were classified for quality grade and stage of development according to the IETS recommendations [63]. Only grade-1 embryos, i.e., embryos consistent with their expected stage of development, with blastomeres uniform in size, color, and density, with few irregularities or excluded cells, and an intact and smooth zona pellucida, were used.

All embryos were thoroughly washed three times in 20 mM Tris–HCl buffer (pH 6.8) supplemented with 8.9% sucrose (Tris-sucrose), minimizing contamination from oviduct and uterine fluids, then individually frozen at -80 °C. Typically, the time period between animal death and genital tract flushing and between flushing and embryo freezing was less than 15 and 30 min, respectively.

### Production of embryos developed *in vitro*

Bovine embryos were produced *in vitro* as previously described [25] in parallel with the flushing sessions. Bovine ovaries were collected from a local slaughterhouse (slaughtering mostly Holstein cows) and transported at 36 °C to the laboratory. Cumulus‐oocyte complexes (COCs) were recovered using HEPES‐buffered TCM-199 supplemented with 0.4 g/L bovine serum albumin (BSA) and 0.25% gentamicin. Groups of 30–60 COCs were matured in TCM-199 supplemented with 10 ng/mL EGF, 19 ng/mL IGF-1, 2.2 ng/mL FGF, 5 UI/mL hCG, 10 UI/mL PMSG, 4 µg/mL transferrin, 4 µg/mL insulin, 5 ng/mL sodium selenite, 1% PG-600, 90 µg/mL L-cysteine, 0.1 mM beta-mercaptoethanol, 75 µg/mL ascorbic acid, 720 µg/mL glycine, 0.1 mg/mL glutamine, and 110 µg/mL pyruvate at 38.8 °C (5% CO2) for 22 h. After maturation, COCs were transferred in 250 µL of fertilization medium (Tyrode medium supplemented with 25 mM bicarbonate, 10 mM lactate, 1 mM pyruvate, 6 mg/mL fatty-acid free BSA, 10 µg/ml heparin, and 40 µg/ml gentamicin). To minimize variability between embryos, the Normande bull X used for most *in vivo*-derived embryos was used for all *in vitro* fertilization (IVF) (Table 1). Motile spermatozoa were recovered by Percoll washing and added to the fertilization medium (day 0) at a final concentration of 10^6^ spermatozoa/mL for 22 h. At day 1, all presumptive zygotes were cultured in 25 µL of synthetic oviductal fluid (SOF) medium [64] supplemented with 0.01% of polyvinyl alcohol (SOF-PVA) under mineral oil (M8410) at 38.8 °C with 5% CO_2_ and 5% O_2_. To avoid protein contamination, embryos were cultured without any serum or protein supplementation. Only grade-1 embryos according to the IETS classification were retained for analysis. As for *in vivo*-derived embryos, all *in vitro*-produced embryos were washed three times in Tris-sucrose then stored individually in 1.5 mL tubes at -80 °C. A total of 9 IVF replicates were done and embryos at a given stage were originated from at least three IVF replicates.

### Analysis of embryo proteins by nanoLC-MS/MS

All embryos (with no sex determination) were thawed on ice and pools of 4 embryos at the same stage after *in vivo* or *in vitro* development were prepared under a stereomicroscope for a total of 3–4 pools per origin × stage (Table 1). Tubes containing embryos were manipulated for no more than 10 min at ambient temperature. After brief pelleting, the excess Tris-sucrose was eliminated and tubes were stored at -80 °C until proteomic analysis. Proteins from pools of embryos were extracted and digested using the PreOmics iST-BCT kit following the manufacturer’s instructions. Briefly, samples were thawed and lysed (denatured, reduced, and alkylated) for 10 min at 95 °C then trypsin/LysC digested for 60 min at 37 °C. Purification of peptides was then carried out at room temperature on spin cartridges and peptides were finally eluted in 10 µL of LC-load buffer. The volume corresponding to one equivalent-embryo (corresponding to approximately 300 ng proteins) was then loaded on a 75 µm × 250 mm IonOpticks Aurora 2 C18 column (Ion Opticks Pty Ltd., Bundoora, Australia). Peptide analysis was performed as previously described [25]. A gradient of basic reversed-phase buffers (Buffer A: 0.1% formic acid, 98% H_2_O MilliQ, 2% acetonitrile; Buffer B: 0.1% formic acid, 100% acetonitrile) was run on a NanoElute high performance liquid chromatography (HPLC) System (Bruker Daltonik GmbH, Bremen, Germany) at a flow rate of 400 nL/min at 50 °C. The liquid chromatography (LC) run lasted for 120 min (2% to 15% of buffer B during 60 min; up to 25% at 90 min; up to 37% at 100 min; up to 95% at 110 min, and finally 95% for 10 min to wash the column). The column was coupled online to a trapped ion motility spectrometry-time of flight (TIMS-TOF) Pro (Bruker Daltonik GmbH, Bremen, Germany) with a CaptiveSpray ion source (Bruker Daltonik). The temperature of the ion transfer capillary was set at 180 °C. Ions were accumulated for 114 ms, and mobility separation was achieved by ramping the entrance potential from -160 V to -20 V within 114 ms. The acquisition of the MS and MS/MS mass spectra was done with average resolutions of 60,000 and 50,000 full width at half maximum (mass range 100–1,700 m*/z*), respectively. To enable the parallel accumulation-serial fragmentation (PASEF) method, precursor *m/z* and mobility information was first derived from full scan TIMS-MS experiments (with a mass range of 100–1,700 m*/z*). The quadrupole isolation width was set to 2 and 3 Th, and for fragmentation, the collision energies varied between 31 and 52 eV depending on the precursor mass and charge. TIMS, MS operation, and PASEF were controlled and synchronized using the control instrument software OtofControl 5.1 (Bruker Daltonik). LC–MS/MS data were acquired using the PASEF method with a total cycle time of 1.31 s, including 1 TIMS MS scan and 10 PASEF MS/MA scans. The 10 PASEF scans (100 ms each) contained, on average, 12 MS/MS scans per PASEF scan. Ion mobility-resolved mass spectra, nested ion mobility, *m/z* distributions, and summed fragment ion intensities were extracted from the raw data file with DataAnalysis 5.1 (Bruker Daltonik GmbH, Bremen, Germany).

### Protein identification and data validation

Peptides were identified using the Mascot software (version 2.5.1; Matrix Science, 454 London, UK) against the UniProt *Bos taurus* database (May 2019; 23,523 sequences) using its automatic decoy database search to calculate a false discovery rate (FDR). The parameters used for database searches included trypsin as enzyme (one missed cleavage allowed), carbamidomethylcysteine as fixed modification, oxidation of methionine, and N-terminal protein acetylation as variable modifications. Monoisotopic mass was considered, and mass tolerance was set at 15 ppm for MS ions and 0.05 Da for MS/MS ions. Mascot results from the target and decoy databases were incorporated to Scaffold Q + software (version 5.0.1, Proteome Software, Portland, USA, www.proteomesoftware.com) for data validation. Threshold for peptide and protein identification were set to 95.0% as specified by the Peptide Prophet algorithm [65] and the Protein Prophet algorithm [66]. To exclude potential human contaminants from the pool of keratin proteins (KRT 1, 2, 5, 7, 8, 10, 14, 18, 19, 24, 28, 71, and 75), the peptides identified in the *Bos taurus* database were blasted against the *Homo sapiens* homologous sequences. For all keratins, the peptide sequences were specific to *Bos Taurus,* excluding human contaminants.

### Label-free protein quantification and statistical analysis

All proteins containing at least two unique peptides (FDR < 0.01%) were considered for protein quantification. Protein quantification was based on a label-free approach using the spectral counting method, as previously described [67, 68]. The “Spectral Count” quantitative module of Scaffold Q + software (Proteome Software, version 5.0.1) was used. The normalization of spectra among samples was realized in “Scaffold” by adjusting the sum of the selected quantitative values for all proteins within each MS sample to a common value, which was the average of the sums of all MS samples present in the experiment. This was achieved by applying a scaling factor for each sample to each protein or protein group. Thus, the number of NWS were tabulated using experiment-wide protein clusters.

Statistical analysis was performed on proteins quantified with a minimum of 2.0 NWS (mean value of biological replicates) in at least one origin × stage. To obtain an overview of proteomic data, PCA of all samples was carried out using RStudio software (version 1.4.1106) and the FactoMineR package. Analysis of variance (ANOVA) on biological replicates was done using RStudio. The hierarchical clustering of DAPs (ANOVA *p*-value ≤ 0.050) were done using Spearman correlations and the “gplots” package of RStudio. Finally, pairwise comparisons of protein abundance between *in vitro* and *in vivo*-derived embryos at a given stage were evaluated by Student’s t-tests using RStudio. Proteins were considered as differentially abundant with a t-test *p*-value ≤ 0.050, fold-change ratio > 1.5, and a minimum of 2.0 NWS (mean value of biological replicates) for higher abundance.

### Functional analysis of differentially abundant proteins

The overlap of identified proteins between stages and origins was visualized using the jvenn online tool (http://jvenn.toulouse.inra.fr) [69]. Functional analysis of DAPs in the three clusters (ANOVA *p*-value ≤ 0.050) and in pairwise comparisons between origins at a given stage (t-test *p*-value ≤ 0.050) was performed using the corresponding gene lists and Metascape online tool (metascape.org) [70]. Since the *Bos taurus* taxonomy was unavailable in Metascape*,* the *Homo sapiens* genome was used as background, and the gene list was converted in *Homo sapiens* orthologs. For individual and combined gene lists, three successive enrichment analyses were undertaken: 1) pathway enrichment using the GO biological processes and the Kyoto Encyclopedia of Genes and Genomes (KEGG) pathways; 2) functional enrichment using the GO molecular functions; and 3) structural enrichment using the GO cellular components. Terms with a *p*-value < 0.01, a minimum count of 3, and an enrichment factor > 1.5 (the enrichment factor is the ratio between the observed counts and the counts expected by chance) were collected and grouped by Metascape into clusters based on their membership similarities. In histograms presenting the enriched terms, the most statistically significant term within a cluster is chosen by Metascape to represent the cluster.

## Supplementary Information

**Additional file 1: Figure S1.** 

**Additional file 2: Tables S1–S8.** 

## Data Availability

The datasets generated during the current study are available in the ProteomeXchange Consortium via the PRIDE [71] partner repository (www.ebi.ac.uk/pride/archive/login) with the dataset identifier PXD035294 and 10.6019/PXD035294.

## Abbreviations

DAP: Differentially abundant protein
EGA: Embryonic Genome Activation
GO: Gene Ontology
IVF: *in vitro* Fertilization
NWS: Normalized Weighted Spectra
PCA: Principal Component Analysis
