# Supplementary figures and images for: The proteomic analysis of bovine embryos developed in vivo or in vitro reveals the contribution of the maternal environment to early embryo
Source: BMC Genomics. 2022 Dec 19;23:839. doi: 10.1186/s12864-022-09076-5 (PMC9764490; doi:10.1186/s12864-022-09076-5)

**Figure S1.** Representative pictures of embryos produced *in vivo* (A–E) and *in vitro* (A'–E')

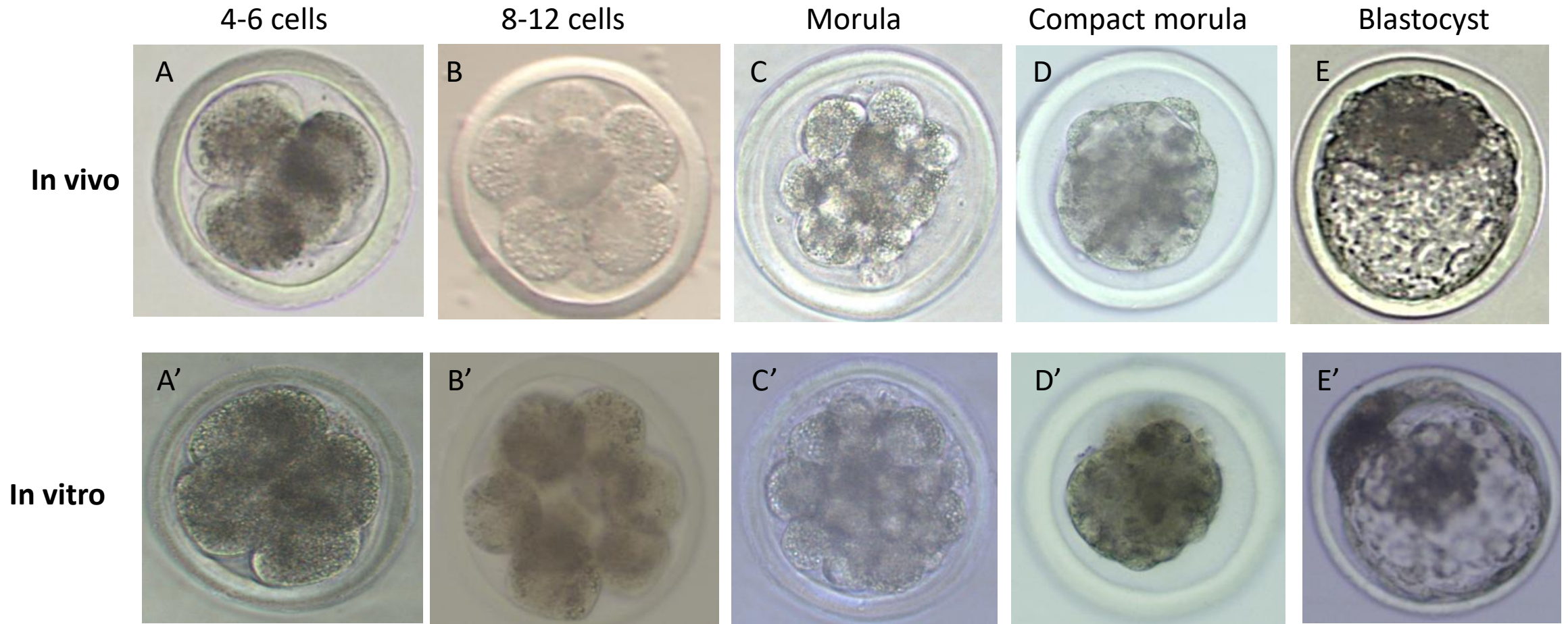

Supplement: Supplementary file 1 — Additional file 1: Figure S1. [file 12864_2022_9076_MOESM1_ESM.pdf]
